# Transition patients: Adults with chronic pediatric disorder presenting to a tertiary pediatric emergency department in Korea

**DOI:** 10.1097/MD.0000000000012346

**Published:** 2018-09-14

**Authors:** Joong Wan Park, Do Kyun Kim, Young Ho Kwak, Jae Yun Jung, Se Uk Lee, So Hyun Paek

**Affiliations:** a Department of Emergency Medicine, Seoul National University Hospital; b Department of Emergency Medicine, Seoul National University College of Medicine, Seoul; c Department of Emergency Medicine, Cha Bundang Medical Center, Seongnam, Gyeonggi-do, Republic of Korea.

**Keywords:** adolescent, chronic disease, emergency medicine, pediatric emergency department, transition to adult care

## Abstract

The primary objective was to evaluate the characteristics of adults (≥18 years old) with chronic pediatric disorders (transition patients) who visited the pediatric emergency department (PED). The secondary objective was to determine the associated factors for their admission.

This study was a retrospective chart review of transition patients seen at a large, urban PED in Korea from 2010 to 2015. Epidemiologic and clinical data were used to identify the characteristics of transition patients compared with those of pediatric patients in the PED. A multivariable logistic regression model was used to calculate odds ratios (ORs) for the factors associated with hospitalization.

Transition patients accounted for 2776 (2.4%) of the total encounters. A total of 463 (38.9%) of the transition patients had >1 visit. Congenital heart disease was the most common (23.2%) chronic pediatric disorder. Most encounters (94.5%) were related to an underlying disorder, and 34.4% of the encounters required consultations with other pediatric subspecialties. Diagnostic procedures were performed in 90.1% of the encounters. Transition patients were hospitalized more than pediatric patients (35.3% vs 15.3%; *P* < .001). The associated factors for admission in the transition patients were chronic gastrointestinal disorder (adjusted odds ratio [AOR]: 3.76 [95% confidence interval, CI, 2.29–6.16]), complaints related to an underlying disorder (AOR: 3.13 [95% CI, 1.94–5.05]), respiratory complaints (AOR: 2.02 [95% CI, 1.33–3.08]), and infectious complaints (AOR: 1.97 [95% CI, 1.40–2.76]).

A substantial number of transition patients used the PED, and they required a larger amount of resources in the PED. Chronic gastrointestinal disorder, respiratory symptoms, or complaints related to an underlying disorder were the related factors for admission of transition patients.

## 1 Introduction

The survival rate of children suffering from serious congenital or acquired diseases and the number of pediatric patients with chronic diseases have increased with the development of medical technology. As these children survived and became adults, a new cohort of “transition patients” has emerged.^[1–4]^ Adult patients with chronic pediatric illnesses are familiar with the systems, medical services, and pediatricians in children's hospitals due to the contact they had with these hospitals as they aged. Therefore, these adult patients still seek medical services in pediatric emergency departments (PEDs) for their acute care problems.^[5]^ PEDs were originally intended to care for acutely ill or injured children. However, PEDs also provide medical care for adult patients with chronic childhood illnesses who have not yet transitioned to an adult medical system.^[6]^ McDonnell et al reported that these transition patients required a large amount of diagnostic and therapeutic resources relative to their numbers and that their hospitalization rates, intensive care unit admission rates, and lengths of hospital stay were increased compared with those of other pediatric patients.^[7]^

Previous studies of adult patients in the PED mostly focused on adult patients not followed by pediatric subspecialists who visited PEDs.^[8–10]^ Although several studies have focused on adult patients who have not transitioned to adult medical care and are still cared for in children's hospitals for underlying medical disorders,^[5,7,11]^ no reports have investigated these transition patients in Korea. In addition, no studies have examined the factors associated with hospitalization in transition patients. To provide appropriate emergency medical care to transition patients, we should obtain a clear picture of the current use of PEDs by transition patients.

The aims of this study were to evaluate the characteristics of transition patients who visited the PEDs in Korea and to analyze factors related to the hospital admission of transition patients.

## 2 Methods

### 2.1 Study design and patients

This study was conducted as a retrospective chart review of all adult patients (defined as 18 years and older) who presented to our children's hospital PED from January 1, 2010 to December 31, 2015. Our hospital is a large, urban, tertiary care, academic children's hospital with an annual ED volume of >20,000 patient encounters. The hospital is nationally recognized as a referral facility for multiple medical subspecialties and is located next to an adult hospital. The patients visiting the PED are generally under 18 years old.

### 2.2 Data and variables

Electronic medical records were queried to identify all visits for adult patients with chronic pediatric disorders. One researcher reviewed all medical charts and abstracted data, including the age at the time of the PED visit, sex, the presence of a chronic pediatric disorder, pediatric subspecialty clinics with ongoing relationships, number of PED visits within the study period, dates and times of the PED admission and discharge, use of emergency medical services at PED presentation, routes of PED presentation, triage acuity, chief complaint at PED presentation, association between chief complaint and underlying disorder, PED diagnostic procedures, other subspecialty consultations in the PED, disposition after emergency care, discharge diagnosis, length of PED stay, and duration of hospital admission. The number of annual PED visits for transition patients was also extracted to identify PED use trends for transition patients.

We relied on narrative descriptions in the medical records rather than codes from the International Classification of Diseases, Tenth Revision (ICD-10), to determine the diagnoses. Triage acuity was assessed using the Emergency Severity Index (ESI). A trained registered nurse assigned each patient to a category ranging from 1 (most urgent) to 5 (least urgent) based on a protocol. No formal definition of a chronic pediatric disorder is available.^[12,13]^ However, the most useful definitions are based on the presence of any functional impairment.^[12]^ Therefore, we defined a chronic pediatric disorder as a medical diagnosis with functional impairment in childhood that persists for ≥1 year and requires continuing medical care.^[14]^ Chronic pediatric disorders were categorized into 23 chronic disorders according to the consensus of pediatric emergency specialists and a previous study.^[7]^ We selected an age of 18 years and older as our cutoff age for adult patients because our PED routinely sees patients until their 18th birthday. Similarly, we defined pediatric subspecialty clinics with ongoing relationships as pediatric subspecialty clinics visited by the patients >1 time within 1 year before the date of the visit to the ED with scheduled continuous follow-up. The presenting complaints were grouped based on 13 organ systems according to the consensus of pediatric emergency specialists and a previous study.^[7]^ Presenting complaints not included in the predefined categories were grouped as “others” (e.g., “for examination” and “for laboratory test”). The association between the presenting complaints and the underlying chronic illness was defined as “yes” if the transition patients consulted their primary pediatric subspecialty at the time of the PED visit. Diagnostic procedures included radiographic studies, laboratory tests, or medical or surgical procedures.

The primary outcomes were the clinical characteristics of the transition patients who visited the PED. To estimate the burden and the severity of the transition patients, we compared transition patients with pediatric patients (defined as all PED patients <18 years of age). Factors influencing the admission of the transition patients were assessed as the secondary outcome.

### 2.3 Statistical analysis

All data calculations were performed using STATA version 14.2 (StataCorp LP, College Station, TX). Continuous variables are presented as medians with ranges or interquartile ranges (IQR; 25th and 75th percentiles). Categorical variables are presented as frequencies with proportions. The χ^2^ test was used to analyze the trend in the number of transition patient visits over time using the number of transition patients and the total number of patient visits during each calendar year. The χ^2^ or Wilcoxon rank-sum test was performed during the univariate analysis of the clinical characteristics between the transition and pediatric patients.

We used multiple logistic regression with hospital admission as the outcome of interest. To detect multicollinearity for independent variables, we used the variance inflation factor (VIF) and Cramer's V. Odds ratios (ORs) > 1 were indicative of a risk of hospital admission. The ORs and 95% confidence intervals (CIs) were derived for all covariates. *P* < .05 was considered significant.

### 2.4 Ethics statement

We obtained approval from our hospital institutional review board before beginning this study (IRB No. 1607-190-781).

## 3 Results

### 3.1 Demographics and clinical characteristics of the transition patients

A total of 115,038 visits to our PED were recorded during the 6-year study period. Of these visits, 3,032 (2.6%) were patients 18 years or older. Of this group of 3,032 adult patient visits, 2,776 (91.6%) were transition patients still followed by pediatric specialists for a variety of chronic disorders. A group of 1,189 transition patients accounted for these 2,776 visits, with frequencies ranging from a minimum of 1 to a maximum of 83 visits per patient. In total, 463 (38.9%) transition patients had >1 visit. Transition patients followed by hematology and oncology tended to visit the PED more frequently. The median age of the transition patients at the time of the visit was 20 years, with a range from 18 to 49 years. Males constituted 56.5% of all transition patients. The most common primary pediatric subspecialty clinic with which transition patients had an ongoing relationship was cardiology (24.1%), followed by neurology (16.7%) and hematology and oncology (12.6%). The median number of pediatric subspecialty clinics with which transition patients had ongoing relationships was 1, with a range from 1 to 6. In total, 2,622 (94.5%) transition patients visited the PED for complaints related to their own underlying chronic pediatric disorder. Of the transition patients who visited the PED, 954 (34.4%) patient visits consulted other pediatric subspecialties in addition to the primary pediatric subspecialty. Diagnostic procedures were performed in 2,499 (90.1%) transition patient visits (Table 1).

**Table 1:**
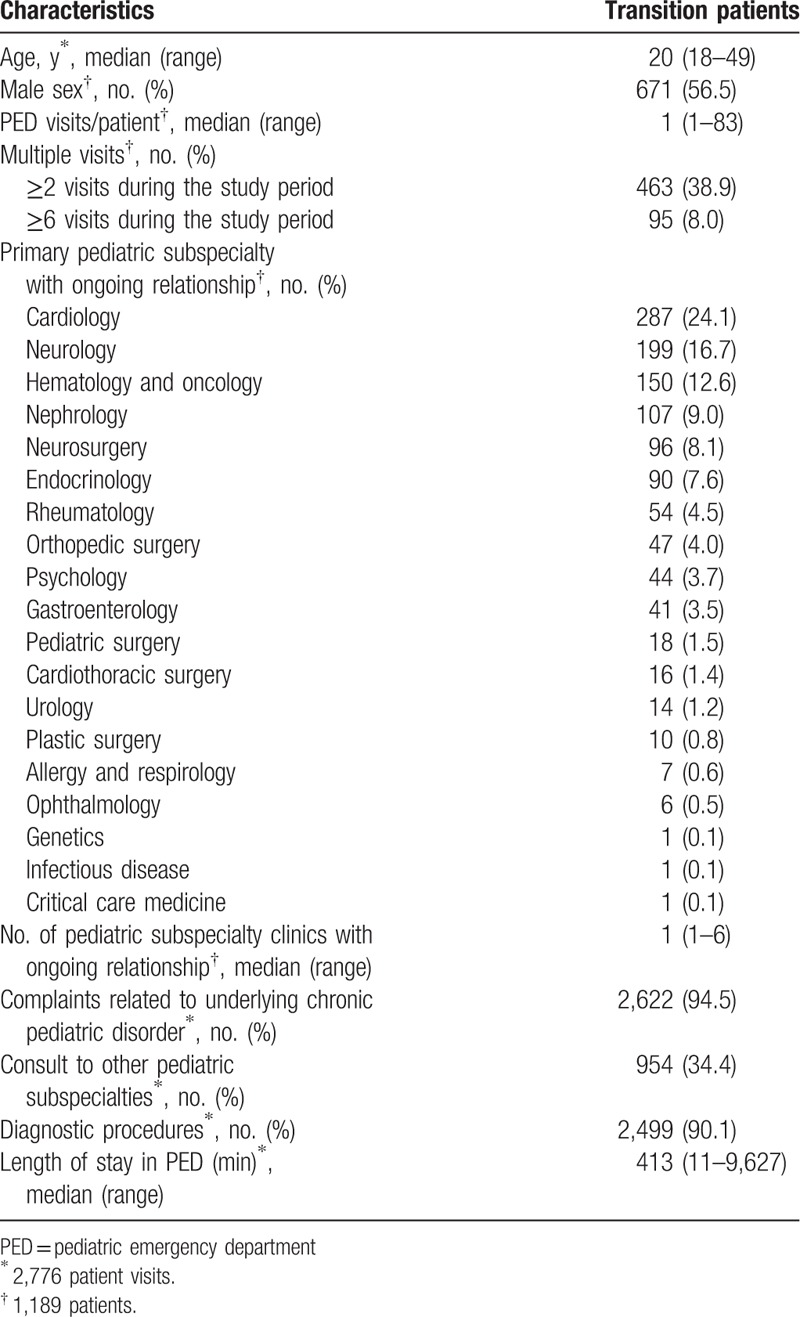
Demographic and clinical characteristics of transition patients.

**Figure 1:**
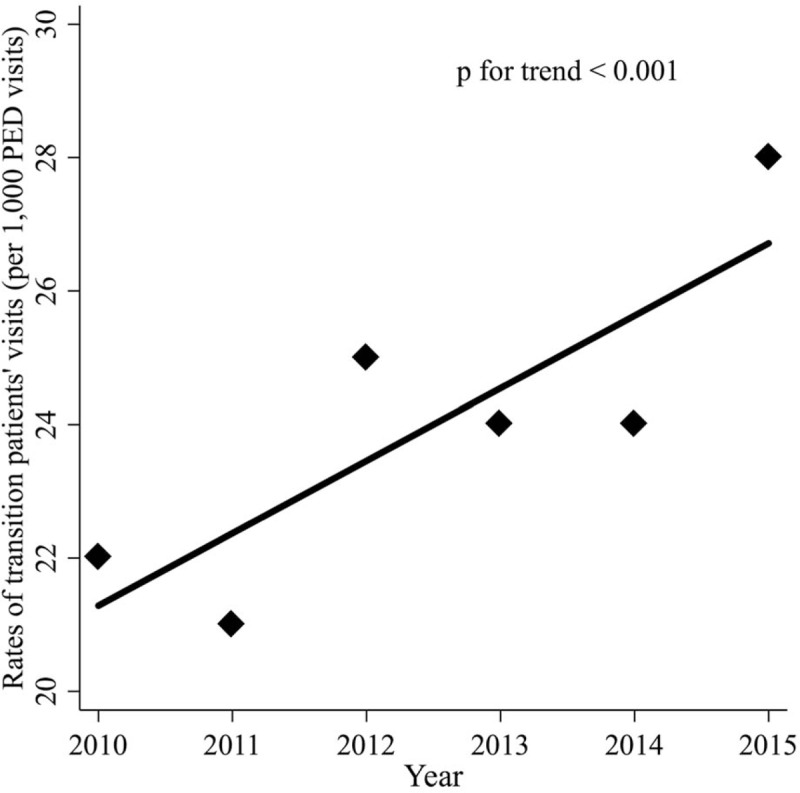
Rate of transition patients’ visits by year (per 1,000 PED visits). PED visits of transition patients exhibited an increasing trend.

The PED visits of transition patients exhibited an increasing trend throughout the study period as follows: 22 (2010), 21 (2011), 25 (2012), 24 (2013), 24 (2014), and 28 (2015) per 1,000 PED visits (*P* for trend <.001) (Fig. 1).

**Figure 2:**
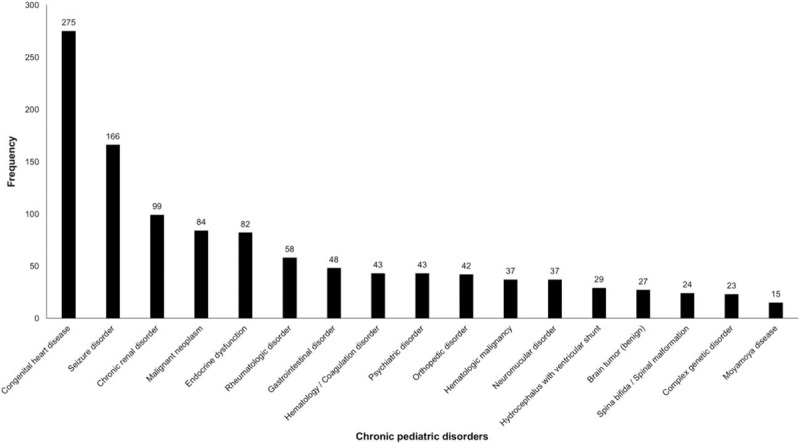
Underlying chronic pediatric disorders of transition patients. Congenital heart disease was the most common chronic pediatric disorder of transition patients.

The transition patients who visited the PED during the study period had a broad variety of chronic pediatric disorders. Congenital heart disease was the most common chronic pediatric disorder, followed by seizure disorders (Fig. 2).

**Figure 3:**
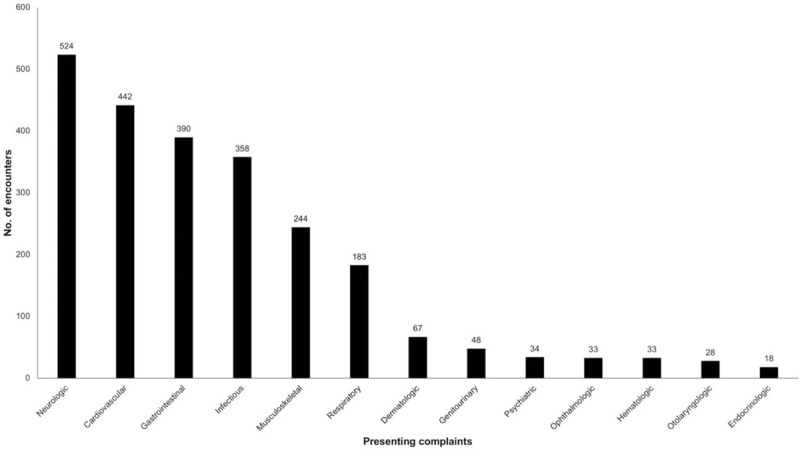
Presenting complaints of transition patients based on organ systems. Neurologic symptoms were the most common presenting complaint of transition patients.

Transition patients who visited the PED during the study period presented with variable complaints. Neurologic symptoms were the most common presenting complaint, followed by cardiovascular, gastrointestinal, and infectious symptoms (Fig. 3).

### 3.2 Characteristics of transition patients compared with pediatric patients

Transition patient encounters referred from outpatient clinics were noted in 8.4% of cases, whereas 3.3% of pediatric patient encounters were referred from outpatient clinics. Transition patients who visited the PED used ambulances more frequently than pediatric patients (119 ambulances, 11.2% vs 4.4%; other ambulances including hospital ambulance, private ambulance, and air transport, 2.9% vs 1.4%; *P* < .001). The ESI level of the transition patient encounters at triage was increased compared with the ESI level of the pediatric patient encounters (*P* < .001). The median length of the PED stay was 413 minutes (IQR 234–927) for the transition patient encounters and 167 minutes (IQR 75–360) for the pediatric patient encounters (*P* < .001). After PED treatment, 1,765 (63.6%) transition patient encounters resulted in discharge, and 26 (0.9%) patients were transferred. After PED treatment, 980 (35.3%) transition patient encounters required hospital admission. Among them, 56 (5.7%) patients required intensive care unit (ICU) admission. In comparison, the ICU admission rate for all admitted pediatric patients during the study period was 849 (4.9%). Both the ward and ICU admission rates were increased for the transition patients compared with the pediatric patients (*P* < .001). The transition patients’ median length of hospitalization was 5 days (IQR 3–9) compared with a median of 4 days (IQR 2–8) for the pediatric patients (*P* < .001) (Table 2).

**Table 2:**
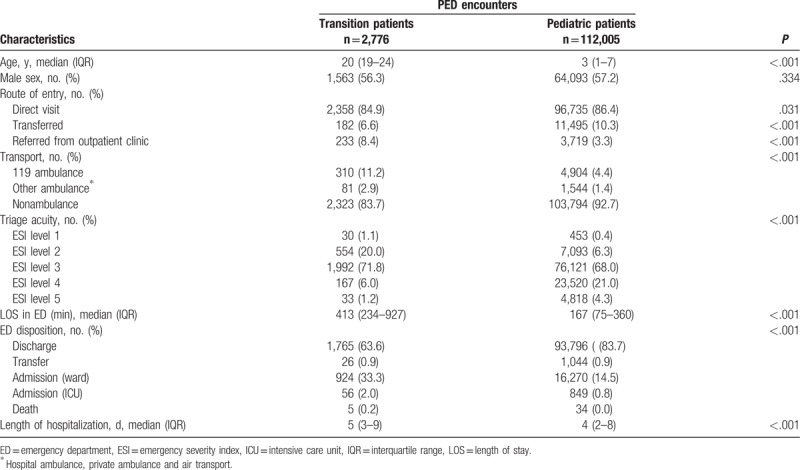
Demographics and clinical characteristics of transition patients compared with pediatric patients.

### 3.3 Factors associated with hospital admission in transition patients

In the univariate analysis, transfer or referral from outpatient clinics, an ambulance mode of transportation to the PED, an ESI level 1 or 2 at triage, underlying neuromuscular or gastrointestinal disorders, gastrointestinal, infectious, or respiratory complaints, complaints related to underlying chronic pediatric disorders, an ongoing relationship with >2 pediatric subspecialty clinics, consultation with another pediatric subspecialty, and undergoing diagnostic procedures were associated with an increased likelihood of hospitalization. Similar to the univariate analysis results, the multiple logistic regression analysis demonstrated that the following variables were associated with the hospital admission of transition patients: transfer (AOR = 2.69 [95% CI, 1.82–3.97]) or referral from outpatient clinics (AOR = 1.86 [95% CI, 1.36–2.54]); an ESI level 1 or 2 at triage (AOR for ESI 1 = 4.65 [95% CI, 1.77–12.20], AOR for ESI 2 = 1.55 [95% CI, 1.24–1.95]); underlying chronic renal disorder (AOR = 1.45 [95% CI, 1.03–2.04]); gastrointestinal disorder (AOR = 3.76 [95% CI, 2.29–6.16]); gastrointestinal complaint (AOR = 1.65 [95% CI, 1.16–2.36]); infectious complaint (AOR = 1.97 [95% CI, 1.40–2.76]); respiratory complaint (AOR = 2.02 [95% CI, 1.33–3.08]); complaints related to an underlying chronic pediatric disorder (AOR = 3.13 [95% CI, 1.94–5.05] ); ongoing relationship with >2 pediatric subspecialty clinics (AOR = 1.37 [95% CI, 1.13–1.67]); consultation with other pediatric subspecialties (AOR = 1.44 [95% CI, 1.18–1.74] ); and receipt of diagnostic procedures (AOR = 4.08 [95% CI, 2.52–6.60]) (Table 3).

**Table 3:**
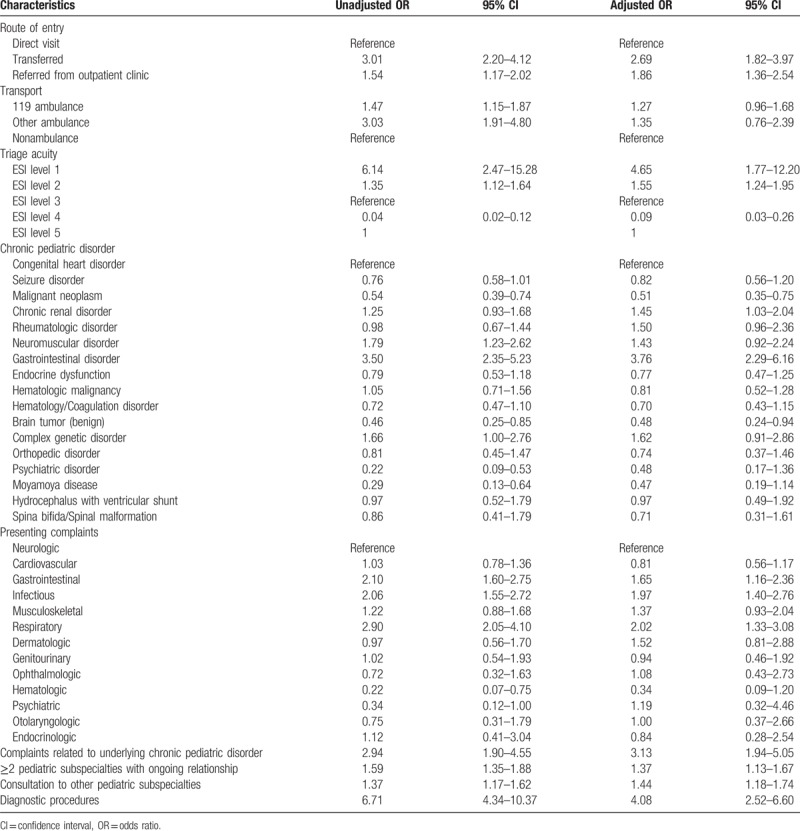
Odds ratios for risk factors associated with admission by univariate analysis and multivariate-adjusted analyses.

## 4 Discussion

This study evaluated the clinical characteristics and associated factors of hospital admission of transition patients in Korea. During the 6-year study period, transition patients accounted for 2.4% of all PED visits and represented a growing patient population. Our study demonstrated that transition patients with various chronic pediatric disorders visited the PED with a broad variety of complaints. In addition, our results indicated that transition patients were more likely to be hospitalized when they had chronic gastrointestinal disorders or visited the PED for respiratory, infectious, or gastrointestinal symptoms.

In our study, the prevalence of transition patient visits to the PED was >0.5% reported by McDonnell et al and 0.04% reported by Little et al.^[7,10]^ One possible explanation for this finding is that our hospital is a national referral facility for multiple medical subspecialties. Our hospital is the oldest children's hospital in Korea with over 30 years of practice, and few Korean children's hospitals can treat complex chronic pediatric illnesses. In addition, because we defined adult patients as those over 18 years of age, more visits were enrolled from adult patients with a relatively young age compared with those in previous studies.^[7–11]^

In accordance with a previous study,^[7]^ transition patients with congenital heart disease comprised the largest subgroup, and the most common presenting complaint of the transition patient was neurological symptoms. A total of 94.5% of the transition patients presented to the PED for medical complaints related to their underlying chronic pediatric disorders in our study, whereas a previous study reported that 55% of PED encounters were based on medical complaints directly related to the chronic pediatric disorder.^[7]^ The use of different definitions of complaints related to chronic pediatric disorders may have contributed to the observed differences. We defined the transition patients’ complaint as related to their chronic pediatric disorder when their primary subspecialty in the PED was consulted. Thus, the high correlation between the presenting complaints and the chronic pediatric illness can be explained.

In this study, one-third of the transition patients consulted other pediatric subspecialties, and most transition patients received diagnostic procedures at the time of the PED visit. A previous study also reported that 86% of transition patients required consultation with ≥1 pediatric subspecialty service and that only 7% of transition patient PED visits did not involve radiographic studies, laboratory tests, or medical or surgical procedures.^[7]^ These findings indicate that transition patients demand substantial resources in the PED. Consistent with previous studies,^[7,9–11]^ our study demonstrated that transition patients in PEDs had relatively high triage acuity and that the duration of the PED stay was longer for the transition patients than for the pediatric patients. This study also demonstrated that the rates of hospitalization and intensive care after PED treatment were increased in transition patients compared with pediatric patients, which was in accordance with a previous study.^[7]^ These findings suggest that transition patients are more likely to be in a more serious medical state when visiting the PED than pediatric patients. Therefore, PEDs must be prepared with experts to care for the acute medical problems of chronic pediatric disorders.

This study has several limitations. First, this study is retrospective in nature. Data concerning the chief complaints for seeking emergent care were limited to the electronic medical record. To overcome this limitation, one researcher reviewed all charts to provide consistency in data interpretation. Second, we only evaluated patient visits at one PED. Therefore, our findings for transition patients may not be generalizable to the entire transition patient population. However, our hospital is a representative children's hospital and the oldest children's hospital in Korea. Therefore, our results may be considered representative of the characteristics of transition patients in Korea. The generalizability of our results may be limited by differing PED use patterns and admitting practices across various healthcare systems and geographic areas. Further studies in other countries and other tertiary hospitals in Korea are needed to clarify the general characteristics of transition patients.

In conclusion, we found that substantial numbers of transition patients used the PED. These patients required a substantial amount of resources in the PED, and the severity of their illnesses was greater than that of the pediatric patients. Chronic gastrointestinal disorder and respiratory complaints are independent risk factors of hospital admission of these patients. PEDs should be equipped with expertise to best care for these patients and should carefully consider the proper allocation of PED resources.

## Author contributions

**Conceptualization:** Joong Wan Park, Do Kyun Kim.

**Data curation:** Joong Wan Park, Do Kyun Kim, Young Ho Kwak, Se Uk Lee, So Hyun Paek.

**Formal analysis:** Joong Wan Park, Jae Yun Jung.

**Project administration:** Do Kyun Kim.

**Supervision:** Do Kyun Kim, Young Ho Kwak, Se Uk Lee, So Hyun Paek.

**Writing – original draft:** Joong Wan Park.

**Writing – review and editing:** Joong Wan Park, Do Kyun Kim, Young Ho Kwak, Jae Yun Jung, Se Uk Lee, So Hyun Paek.
